# Low prognostic nutritional index score is associated with lymph node metastasis in patients with breast cancer, whereas hemoglobin-albumin-lymphocyte-platelet score is not

**DOI:** 10.3389/fonc.2026.1780492

**Published:** 2026-04-27

**Authors:** Weihua Wen, Litao Jin, Yuyang Yang, Jianjuan Li

**Affiliations:** Department of Breast Surgery, Meizhou People's Hospital, Meizhou Academy of Medical Sciences, Meizhou, China

**Keywords:** biomarker, breast cancer, hemoglobin-albumin-lymphocyte-platelet score, lymph node metastasis, prognostic nutritional index score

## Abstract

**Background:**

Nutritional status and immune function are pivotal to the progression of cancer. The present study is designed to explore the associations between the prognostic nutritional index (PNI) score, hemoglobin-albumin-lymphocyte-platelet (HALP) score, and lymph node metastasis (LNM) in breast cancer patients.

**Methods:**

A total of 799 breast cancer patients were enrolled in this retrospective study, and their clinical data were collected. The PNI and HALP score were calculated for all patients. According to the status of LNM, the patients were divided into LNM-positive patients and LNM-negative cohort. The differences in PNI and HALP score between the two groups were compared, and statistical analysis was performed to clarify the relationship between these two scores and LNM.

**Results:**

412 cases (51.6%) were confirmed with LNM, whereas the remaining 387 (48.4%) were LNM-free. The median levels of both HALP (37.9 (28.2, 46.8) vs. 42.0 (33.4, 54.6), *p* < 0.001) and PNI (51.2 (48.3, 53.8) vs. 52.6 (50.2, 55.3), *p* < 0.001) were notably lower in patients with LNM than in those without this condition. When LNM was designated as the endpoint for receiver operating characteristic (ROC) curve analysis of HALP and PNI levels, the optimal cutoff values were determined to be 39.95 for HALP and 52.45 for PNI. Logistic regression analysis showed that low PNI levels (odds ratio [OR]: 1.481, 95% confidence interval [CI]: 1.016-2.159, *p* = 0.041) was associated with LNM, but HALP not (*p* = 0.257).

**Conclusions:**

Low PNI is a potential risk factor for LNM in breast cancer. Clinically, PNI can be used as a convenient warning tool for LNM in breast cancer, and attention should be paid to nutritional and immune intervention before and after surgery.

## Introduction

Breast cancer is one of the most common malignant tumors among women worldwide (1). In terms of incidence and mortality rates, the disease burden of breast cancer is increasing steadily (2). In China, its disease burden is also growing, and the trend of younger onset cannot be ignored (3, 4). Lymph node metastasis (LNM) of breast cancer refers to a pathological process in which breast cancer cells detach from the primary tumor focus, invade the regional or distant lymph nodes via the lymphatic circulation, and proliferate and grow within the lymph nodes to form metastatic tumor lesions (5, 6). As an important prognostic factor for breast cancer, LNM is closely associated with postoperative recurrence, distant metastasis risk, and the survival rate of patients (7–11). Identifying the risk factors for LNM is of great clinical significance for optimizing the individualized treatment plans for breast cancer patients, formulating preoperative assessment strategies, and improving prognosis.

In recent years, the impact of nutritional status and immune function on the progression of malignant tumors has gradually become a research hotspot (12, 13). The occurrence and progression of malignant tumors depend not only on the proliferative capacity of tumor cells themselves but also on the systemic nutritional status and immune dysfunction of the host organism (14, 15). The growth and metastasis of tumors depend on the nutritional supply of the body, and at the same time, they can disrupt the internal environment balance of the host by consuming a large amount of nutrients and inhibiting the function of immune cells, forming a vicious cycle of “malnutrition - immunosuppression - tumor progression” (16, 17). Against this background, a series of nutritional immune scoring systems based on hematological indicators have emerged. These scoring systems, due to their advantages of convenient detection, low cost, and strong repeatability, have been widely used in the prognosis assessment of tumor patients (18, 19). A variety of nutritional and immune−related indicators have been widely applied to evaluate the prognosis and clinicopathological characteristics of malignant tumors, including the Nutritional Risk Index (NRI) (20), Pan-Immune-Inflammatory Value (PIV) (21), Systemic Immune-Inflammation Index (SII) (22), Neutrophil−to-Lymphocyte Ratio (NLR) (23), Platelet−to-Lymphocyte Ratio (PLR) (24), and other systemic immune−inflammatory indices (25). However, some of these indicators only reflect a single aspect of nutritional or inflammatory status, and few of them comprehensively integrate both nutritional and immune conditions.

Prognostic nutritional index (PNI) and the hemoglobin-albumin-lymphocyte-platelet (HALP) score are two commonly used indicators in clinical research in recent years. The PNI assesses the nutritional-immune status of patients by quantifying serum albumin levels and peripheral blood lymphocyte counts. Numerous studies have confirmed that low PNI levels are closely associated with poor prognosis in patients with gastric cancer, colorectal cancer, and lung cancer (26–28). The HALP score integrates four indicators: hemoglobin, albumin, lymphocytes, and platelets. By comprehensively assessing the body’s nutritional, immune, and inflammatory levels, it provides a reference for prognosis judgment of cancer patients (29, 30). However, the relationship between the PNI score, HALP score and LNM in breast cancer remains inconclusive. The present study was designed to evaluate the correlation between these two scores and LNM in breast cancer patients, thereby providing valuable reference data for the diagnosis and treatment regimens of breast cancer patients.

## Materials and methods

### Participants

**Figure 1:**
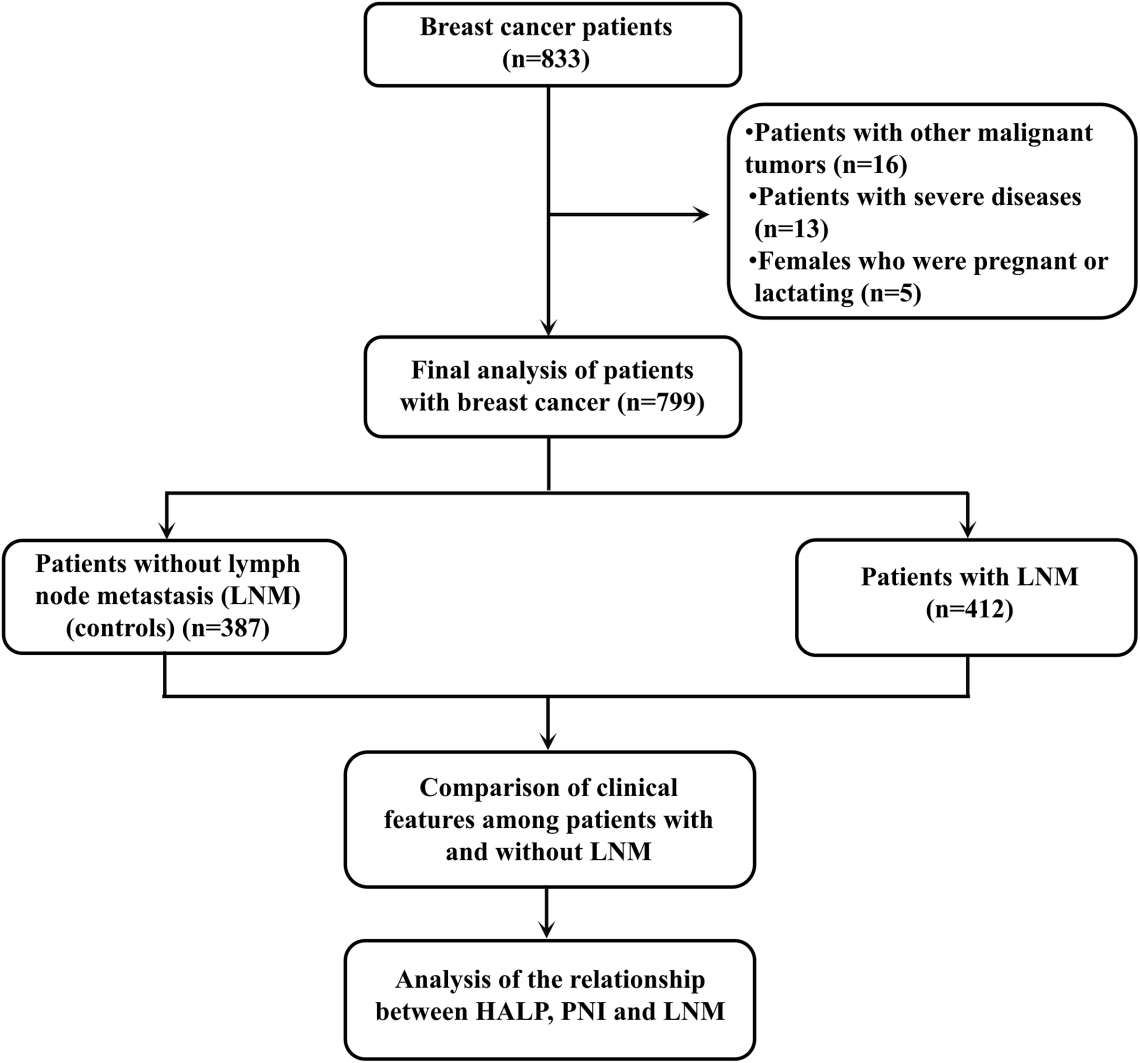
The flowchart of this study.

This investigation adopted a retrospective cohort design, with participants recruited from breast cancer patients who underwent treatment at our hospital between July 2017 and November 2024. Inclusion criteria: (1) confirmed diagnosis of primary breast cancer via histopathological examination; (2) no prior history of antitumor therapies (such as radiotherapy, chemotherapy, or targeted therapy) prior to surgical intervention; and (3) availability of complete clinical and follow-up datasets, encompassing demographic characteristics, pathological profiles, and laboratory assay results. Exclusion criteria: (1) comorbidity with other malignant tumors; (2) concurrent severe diseases including severe infections, autoimmune disorders, hepatic or renal failure, and hematological diseases; (3) females who were pregnant or lactating; and (4) incomplete clinical data that precluded the calculation of PNI and HALP scores as well as the determination of lymph node metastasis status. A total of 799 eligible patients were ultimately enrolled in this study, which has been approved by the Ethics Committee of Meizhou People’s Hospital. The flowchart of this study is shown in **Figure 1**.

### Data collection

Clinical data of the enrolled patients were retrieved from the hospital’s electronic medical record system, encompassing the following categories: (1) demographic characteristics: age and gender; (2) medical history: hypertension history, diabetes mellitus history, and family history of cancer; (3) pathological characteristics: tumor location (left breast, right breast, or bilateral), TNM staging, molecular subtypes (luminal A, luminal B, human epidermal growth factor receptor 2 (HER2)-positive, and triple-negative breast cancer (TNBC)) (31), LNM status, and distant metastasis status; (4) laboratory test results: preoperative serum albumin, hemoglobin, lymphocyte count, and platelet count.

The diagnostic criteria of diabetes mellitus are defined as follows: serum glucose concentration of ≥11.1mmol/L; or fasting blood glucose (FBG) level of ≥7mmol/L; or a 2-hour postprandial glucose concentration of ≥11.1mmol/L measured via an oral glucose tolerance test (32). Diagnostic criteria for hypertension are established as follows: a mean systolic blood pressure (SBP) exceeding 140 mmHg and/or a mean diastolic blood pressure (DBP) greater than 90 mmHg (33). Clinical staging was performed based on the 8th edition of the Tumor-Node-Metastasis (TNM) staging system for breast cancer, which was formulated by the American Joint Committee on Cancer (AJCC) (34).

### Data processing and statistical analysis

The PNI score is calculated based on the serum albumin level (g/L) and the peripheral blood lymphocyte count (×10^9^/L). The calculation formula is as follows: PNI=serum albumin (g/L) + 5 × lymphocyte count (×10^9^/L).

The HALP score is based on hemoglobin (g/L), serum albumin (g/L), lymphocyte count (×10^9^/L) and platelet count (×10^9^/L), and the calculation formula is: HALP = hemoglobin × albumin × lymphocyte count/platelet count.

All data in this study were collated and analyzed using the SPSS statistical software version 26.0 (IBM Inc., USA). Measurement data conforming to a normal distribution were expressed as mean ± standard deviation, while those not conforming to a normal distribution were presented as median (interquartile range (IQR)). Categorical data were described as case number (percentage) [n(%)]. The Mann-Whitney U test was employed for comparisons of measurement data. For categorical variables, either the chi-square (χ^2^) test or Fisher’s exact test was selected for comparative analysis. The diagnostic performance of PNI and HALP for identifying LNM was determined using receiver operating characteristic (ROC) curve analysis. The area under the ROC curve (AUC) was calculated to evaluate the discriminatory ability of PNI and HALP for LNM, and the optimal cutoff values for these two indicators were determined using the Youden index. Logistic regression analysis was conducted to explore the association between HALP, PNI and the risk of LNM. Statistical significance set at *p* < 0.05.

## Results

### Clinicopathological features of patients

A total of 799 breast cancer patients were enrolled in the study, among whom 508 cases (63.6%) were aged ≤ 55 years and 291 cases (36.4%) were older than 55 years. Specifically, 184 patients (23.0%) had a history of hypertension, 93 (11.6%) had diabetes mellitus, and 48 (6.0%) had a family history of cancer. With respect to clinical staging, 233 patients (29.2%) were classified as stage I, 329 (41.2%) as stage II, 177 (22.2%) as stage III, and 58 (7.3%) as stage IV. In terms of molecular subtypes, luminal A, luminal B, HER2-positive, and TNBC accounted for 12.6% (101/799), 29.7% (237/799), 11.8% (94/799), and 11.3% (90/799) of the cohort, respectively. Additionally, the median values of HALP and PNI were 40.0 (30.7, 49.6) and 51.8 (49.3, 54.7), respectively (**Table 1**).

**Table 1:** The clinical features of all patients.

Clinicopathological features	Total (n=799)
Age (years)	
≤55, n (%)	508(63.6%)
>55, n (%)	291(36.4%)
Gender	
Male, n(%)	3(0.4%)
Female, n(%)	796(99.6%)
Hypertension	
No, n(%)	615(77.0%)
Yes, n(%)	184(23.0%)
Diabetes mellitus	
No, n(%)	706(88.4%)
Yes, n(%)	93(11.6%)
Family history of cancer	
No, n (%)	751(94.0%)
Yes, n (%)	48(6.0%)
Laterality of breast cancer	
Left, n (%)	405(50.7%)
Right, n (%)	383(47.9%)
Bilateral, n (%)	11(1.4%)
TNM stage	
I, n (%)	233(29.2%)
II, n (%)	329(41.2%)
III, n (%)	177(22.2%)
IV, n (%)	58(7.3%)
II, III (bilateral breast cancers), n (%)	2(0.3%)
Lymph node metastasis (LNM)	
No, n (%)	387(48.4%)
Yes, n (%)	412(51.6%)
Distant metastasis	
No, n (%)	741(92.7%)
Yes, n (%)	58(7.3%)
Molecular subtypes	
Luminal A, n (%)	101(12.6%)
Luminal B, n (%)	237(29.7%)
HER2+, n (%)	94(11.8%)
TNBC, n (%)	90(11.3%)
Different molecular subtypes of bilateral breast cancers	3(0.3%)
Unknown, n (%)	274(34.3%)
HALP, median (IQR)	40.0 (30.7, 49.6)
PNI, median (IQR)	51.8 (49.3, 54.7)

TNM, tumor-node-metastasis; TNBC, triple-negative breast cancer; HER2, human epidermal growth factor receptor 2; HALP, hemoglobin-albumin-lymphocyte-platelet score; PNI, prognostic nutritional index; IQR, interquartile range.

### Comparison of clinical features among LNM-positive patients and LNM-negative cohort

Of the 799 enrolled patients, 412 cases (51.6%) were confirmed with LNM, whereas the remaining 387 (48.4%) were LNM-free. The prevalence of diabetes mellitus was significantly higher in the LNM-positive group compared with the LNM-negative cohort (13.8% vs. 9.3%, *p* = 0.048). In contrast, the median levels of both HALP (37.9 (28.2, 46.8) vs. 42.0 (33.4, 54.6), *p* < 0.001) and PNI (51.2 (48.3, 53.8) vs. 52.6 (50.2, 55.3), *p* < 0.001) were notably lower in patients with LNM than in those without this condition. No statistically significant between-group differences were observed in terms of age (*p* = 0.106), gender (*p* = 1.000), hypertension status (*p* = 0.110), family history of cancer (*p* = 0.298), tumor laterality (*p* = 0.386), and molecular subtype distribution (*p* = 0.283) (**Table 2**).

**Table 2:** Comparison of clinical features among patients with or without LNM.

Clinicopathological features	Patients without LNM (n=387)	Patients with LNM (n=412)	*p* (χ^2^/Z)
Age (years)			
≤55, n (%)	235(60.7%)	273(66.3%)	0.106 (χ^2^ = 2.644)
>55, n (%)	152(39.3%)	139(33.7%)	
Gender			
Male, n(%)	1(0.3%)	2(0.5%)	1.000 (χ^2^ = 0.275)
Female, n(%)	386(99.7%)	410(99.5%)	
Hypertension			
No, n(%)	288(74.4%)	327(79.4%)	0.110 (χ^2^ = 2.759)
Yes, n(%)	99(25.6%)	85(20.6%)	
Diabetes mellitus			
No, n(%)	351(90.7%)	355(86.2%)	0.048 (χ^2^ = 3.986)
Yes, n(%)	36(9.3%)	57(13.8%)	
Family history of cancer			
No, n (%)	360(93.0%)	391(94.9%)	0.298 (χ^2^ = 1.249)
Yes, n (%)	27(7.0%)	21(5.1%)	
Laterality of breast cancer			
Left, n (%)	205(53.0%)	200(48.5%)	0.386 (χ^2^ = 2.003)
Right, n (%)	178(46.0%)	205(49.8%)	
Bilateral, n (%)	4(1.0%)	7(1.7%)	
TNM stage			
I-II, n (%)	382(98.7%)	180(43.7%)	<0.001 (χ^2^ = 287.606)
III-IV, n (%)	5(1.3%)	232(56.3%)	
Molecular subtypes			
Luminal A, n (%)	53(13.7%)	48(11.7%)	0.283 (χ^2^ = 3.812)
Luminal B, n (%)	102(26.4%)	135(32.8%)	
HER2+, n (%)	37(9.6%)	57(13.8%)	
TNBC, n (%)	40(10.3%)	50(12.1%)	
HALP, median (IQR)	42.0 (33.4, 54.6)	37.9 (28.2, 46.8)	<0.001 (Z=-5.781)
PNI, median (IQR)	52.6 (50.2, 55.3)	51.2 (48.3, 53.8)	<0.001 (Z=-5.578)

TNM, tumor-node-metastasis; TNBC, triple-negative breast cancer; HER2, human epidermal growth factor receptor 2; HALP, hemoglobin-albumin-lymphocyte-platelet score; PNI, prognostic nutritional index; IQR, interquartile range.

### Comparison of clinical features among patients with and without LNM in patients with low and high HALP, respectively

**Figure 2:**
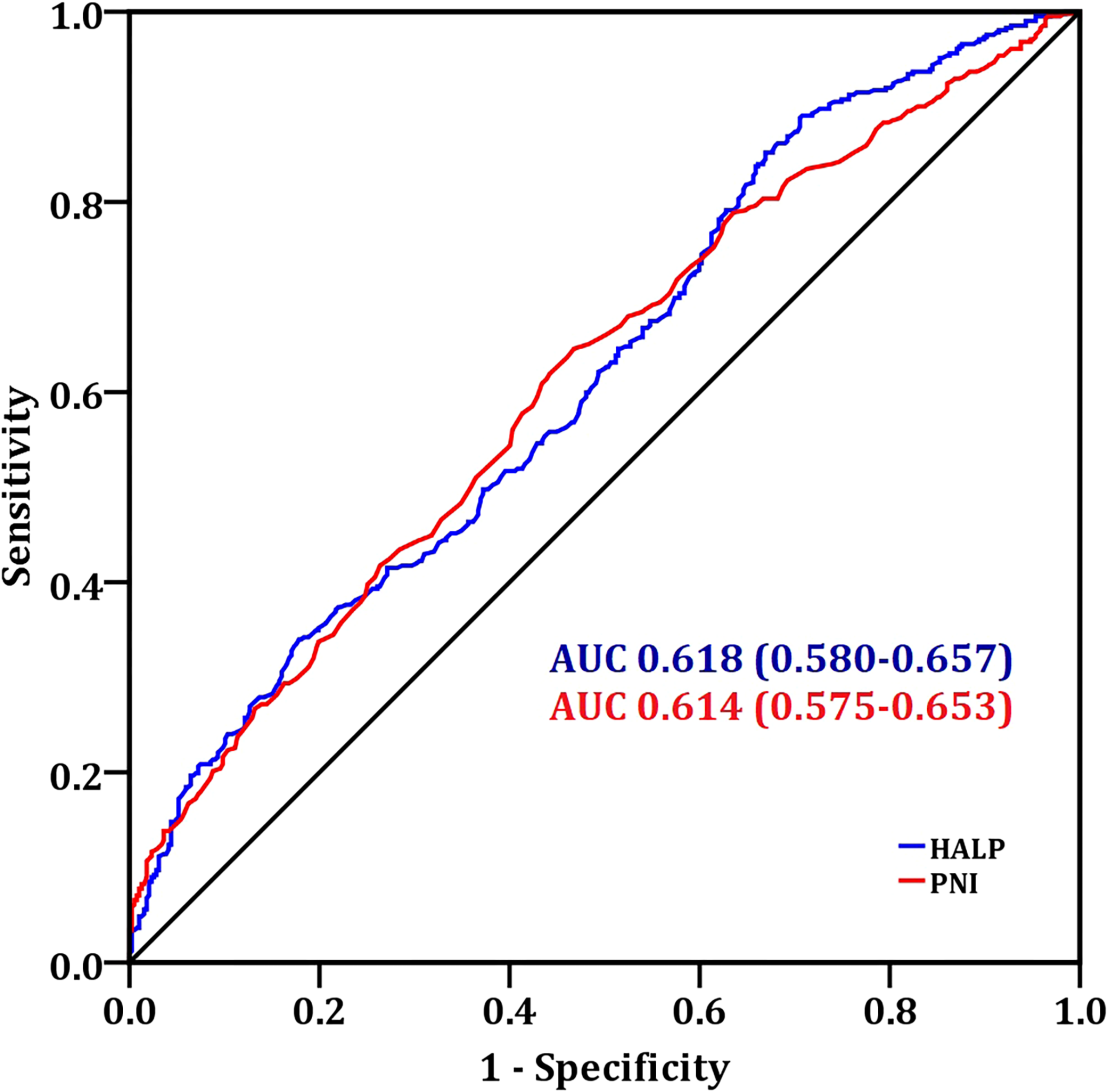
The ROC curve analysis of PNI and HALP to distinguish LNM. PNI, prognostic nutritional index; HALP, hemoglobin-albumin-lymphocyte-platelet score.

The optimal cutoff values of HALP and PNI were 39.95 and 52.45 by ROC analysis, respectively (**Figure 2**). Among patients with HALP <39.95 (n=399), 170 cases (42.6%) were free of LNM, while 229 cases (57.4%) were LNM-positive. No significant between-group differences were detected regarding age (*p* = 0.504), hypertension status (*p* = 0.899), diabetes mellitus (*p* = 0.117), family history of cancer (*p* = 0.150), tumor laterality (*p* = 0.439), or molecular subtypes (*p* = 0.055) (**Table 3**).

**Table 3:** Comparison of clinical features among patients with and without LNM in patients with low and high HALP, respectively.

Clinicopathological features	HALP <39.95 (n=399)			HALP ≥39.95 (n=400)		
	Non-LNM (n=170)	LNM (n=229)	*p* (χ^2^)	Non-LNM (n=217)	LNM (n=183)	*p* (χ^2^)
Age (years)						
≤55, n (%)	124(72.9%)	159(69.4%)	0.504 (χ^2^ = 0.583)	111(51.2%)	114(62.3%)	0.026 (χ^2^ = 5.009)
>55, n (%)	46(27.1%)	70(30.6%)		106(48.8%)	69(37.7%)	
Hypertension						
No, n(%)	135(79.4%)	184(80.3%)	0.899 (χ^2^ = 0.054)	153(70.5%)	143(78.1%)	0.087 (χ^2^ = 3.008)
Yes, n(%)	35(20.6%)	45(19.7%)		64(29.5%)	40(21.9%)	
Diabetes mellitus						
No, n(%)	159(93.5%)	203(88.6%)	0.117 (χ^2^ = 2.765)	192(88.5%)	152(83.1%)	0.148 (χ^2^ = 2.422)
Yes, n(%)	11(6.5%)	26(11.4%)		25(11.5%)	31(16.9%)	
Family history of cancer						
No, n (%)	155(91.2%)	218(95.2%)	0.150 (χ^2^ = 2.588)	205(94.5%)	173(94.5%)	1.000 (χ^2^ = 0.001)
Yes, n (%)	15(8.8%)	11(4.8%)		12(5.5%)	10(5.5%)	
Laterality of breast cancer						
Left, n (%)	84(49.4%)	108(47.2%)	0.439 (χ^2^ = 1.762)	121(55.8%)	92(50.3%)	0.557 (χ^2^ = 1.356)
Right, n (%)	85(50.0%)	116(50.7%)		93(42.9%)	89(48.6%)	
Bilateral, n (%)	1(0.6%)	5(2.2%)		3(1.4%)	2(1.1%)	
TNM stage						
I-II, n (%)	169(99.4%)	89(38.9%)	<0.001 (χ^2^ = 155.689)	213(98.2%)	91(49.7%)	<0.001 (χ^2^ = 126.538)
III-IV, n (%)	1(0.6%)	139(60.7%)		4(1.8%)	91(49.7%)	
Molecular subtypes						
Luminal A, n (%)	31(18.2%)	28(12.2%)	0.055 (χ^2^ = 7.619)	22(10.1%)	20(10.9%)	0.570 (χ^2^ = 2.016)
Luminal B, n (%)	56(32.9%)	78(34.1%)		46(21.2%)	57(31.1%)	
HER2+, n (%)	12(7.1%)	29(12.7%)		25(11.5%)	28(15.3%)	
TNBC, n (%)	12(7.1%)	28(12.2%)		28(12.9%)	22(12.0%)	

TNM, tumor-node-metastasis; TNBC, triple-negative breast cancer; HER2, human epidermal growth factor receptor 2; HALP, hemoglobin-albumin-lymphocyte-platelet score.

Among patients with HALP ≥39.95 (n=400), 217 cases (54.3%) were LNM-negative, and 183 cases (45.7%) were LNM-positive. The proportion of young patients was significantly higher in the LNM-positive subgroup than in the LNM-negative subgroup (62.3% vs. 51.2%, *p* = 0.026). No statistically significant differences were observed between the two groups with respect to hypertension status (*p* = 0.087), diabetes mellitus (*p* = 0.148), family history of cancer (*p* = 1.000), tumor laterality (*p* = 0.557), and molecular subtype distribution (*p* = 0.570) (**Table 3**).

### Comparison of clinical features among patients with and without LNM in patients with low and high PNI, respectively

Among patients with PNI <52.45 (n=447), 181 cases (40.5%) were LNM-negative, while 266 cases (59.5%) were LNM-positive. No significant differences were detected in age (*p* = 0.135), hypertension status (*p* = 0.244), diabetes mellitus (*p* = 0.292), tumor laterality (*p* = 0.557), or molecular subtypes (*p* = 0.061) between patients with and without LNM (**Table 4**).

**Table 4:** Comparison of clinical features among patients with and without LNM in patients with low and high PNI, respectively.

Clinicopathological features	PNI <52.45 (n=447)			PNI ≥52.45 (n=352)		
	Non-LNM (n=181)	LNM (n=266)	*p* (χ^2^)	Non-LNM (n=206)	LNM (n=146)	*p* (χ^2^)
Age (years)						
≤55, n (%)	106 (58.6%)	175 (65.8%)	0.135 (χ^2^ = 2.409)	129 (62.6%)	98 (67.1%)	0.429 (χ^2^ = 0.756)
>55, n (%)	75 (41.4%)	91 (34.2%)		77 (37.4%)	48 (32.9%)	
Hypertension						
No, n(%)	136 (75.1%)	213 (80.1%)	0.244 (χ^2^ = 1.534)	152 (73.8%)	114 (78.1%)	0.380 (χ^2^ = 0.854)
Yes, n(%)	45 (24.9%)	53 (19.9%)		54 (26.2%)	32 (21.9%)	
Diabetes mellitus						
No, n(%)	164 (90.6%)	232 (87.2%)	0.292 (χ^2^ = 1.224)	187 (90.8%)	123 (84.2%)	0.068 (χ^2^ = 3.467)
Yes, n(%)	17 (9.4%)	34 (12.8%)		19 (9.2%)	23 (15.8%)	
Family history of cancer						
No, n (%)	165 (91.2%)	257 (96.6%)	0.020 (χ^2^ = 6.073)	195 (94.7%)	134 (91.8%)	0.382 (χ^2^ = 1.160)
Yes, n (%)	16 (8.8%)	9 (3.4%)		11 (5.3%)	12 (8.2%)	
Laterality of breast cancer						
Left, n (%)	96 (53.0%)	129 (48.5%)	0.557 (χ^2^ = 1.172)	109 (52.9%)	71 (48.6%)	0.689 (χ^2^ = 0.696)
Right, n (%)	83 (45.9%)	132 (49.6%)		95 (46.1%)	73 (50.0%)	
Bilateral, n (%)	2 (1.1%)	5 (1.9%)		2 (1.0%)	2 (1.4%)	
TNM stage						
I-II, n (%)	179 (98.9%)	106 (39.8%)	<0.001 (χ^2^ = 161.713)	203 (98.5%)	74 (50.7%)	<0.001 (χ^2^ = 115.448)
III-IV, n (%)	2 (1.1%)	159 (59.8%)		3 (1.5%)	71 (48.6%)	
Molecular subtypes						
Luminal A, n (%)	31 (17.1%)	26 (9.8%)	0.061 (χ^2^ = 7.381)	22 (10.7%)	22 (15.1%)	1.000 (χ^2^ = 0.017)
Luminal B, n (%)	60 (33.1%)	94 (35.3%)		42 (20.4%)	41 (28.1%)	
HER2+, n (%)	15 (8.3%)	36 (13.5%)		22 (10.7%)	21 (14.4%)	
TNBC, n (%)	19 (10.5%)	30 (11.3%)		21 (10.2%)	20 (13.7%)	

TNM, tumor-node-metastasis; TNBC, triple-negative breast cancer; HER2, human epidermal growth factor receptor 2; PNI, prognostic nutritional index.

Among patients with PNI ≥52.45 (n=352), 206 cases (58.5%) were LNM-negative, whereas 146 cases (41.5%) were LNM-positive. No statistically significant differences were identified between the two subgroups with respect to age (*p* = 0.429), hypertension status (*p* = 0.380), diabetes mellitus (*p* = 0.068), family history of cancer (*p* = 0.382), tumor laterality (*p* = 0.689), and molecular subtype distribution (*p* = 1.000) (**Table 4**).

### Logistic regression analysis of the relationship between HALP, PNI and LNM in breast cancer patients

Univariate analysis results revealed that diabetes mellitus (odds ratio [OR]: 1.565, 95% confidence interval [CI]: 1.006-2.437, *p* = 0.047), low HALP levels (OR: 1.597, 95% CI: 1.208-2.112, *p* = 0.001), and low PNI levels (OR: 2.074, 95% CI: 1.564-2.754, *p* < 0.001) were significantly correlated with LNM in breast cancer patients. Following multivariate logistic regression analysis, diabetes mellitus (OR: 1.884, 95% CI: 1.039-3.415, *p* = 0.037) and low PNI levels (OR: 1.481, 95% CI: 1.016-2.159, *p* = 0.041) remained independently associated with LNM (**Table 5**).

**Table 5:** Logistic regression analysis of the relationship between HALP, PNI and LNM.

Variables	Univariate		Multivariate	
	OR (95% CI)	*P* values	OR (95% CI)	*P* values
Age (>55 vs. ≤55, years old)	0.787 (0.590-1.051)	0.104	0.719 (0.487-1.062)	0.097
Hypertension (yes vs. no)	0.756 (0.543-1.052)	0.097	0.927 (0.574-1.499)	0.758
Diabetes mellitus (yes vs. no)	1.565 (1.006-2.437)	0.047	1.884 (1.039-3.415)	0.037
Family history of cancer (yes vs. no)	0.716 (0.398-1.289)	0.266	0.844 (0.424-1.678)	0.629
Laterality of breast cancer (right vs. left)	1.180 (0.892-1.562)	0.245	1.192 (0.833-1.706)	0.336
Molecular subtypes (non-luminal vs. luminal)	1.172 (0.816-1.683)	0.391	1.321 (0.907-1.925)	0.147
HALP (<39.95 vs. ≥39.95)	1.597 (1.208-2.112)	0.001	1.245 (0.853-1.816)	0.257
PNI (<52.45 vs. ≥52.45)	2.074 (1.564-2.754)	<0.001	1.481 (1.016-2.159)	0.041

HALP, hemoglobin-albumin-lymphocyte-platelet score; PNI, prognostic nutritional index; OR, odds ratio; CI, confidence interval.

To further ensure the robustness of the results, we analyzed the associations of HALP and PNI LNM in breast cancer patients stratified by different molecular subtypes. The main findings remained consistent: PNI was likely associated with LNM in Luminal A, HER2+, and TNBC subtypes, and showed a borderline significant association with LNM in Luminal B breast cancer (*p* = 0.052); in contrast, HALP exhibited no significant correlation with LNM across all molecular subtypes of breast cancer (**Table 6**).

**Table 6:** Logistic regression analysis of the association of HALP, PNI with LNM in patients with different molecular subtypes.

Molecular subtypes	Variables	Adjusted OR (95% CI)	*P* values
Luminal A			
	HALP	1.024 (0.438-2.395)	0.956
	PNI	1.286 (1.051-1.461)	0.048
Luminal B			
	HALP	1.152 (0.676-1.962)	0.602
	PNI	1.743 (1.096-3.048)	0.052
HER2+			
	HALP	2.004 (0.817-4.913)	0.129
	PNI	2.428 (1.002-5.879)	0.049
TNBC			
	HALP	1.732 (0.710-4.230)	0.228
	PNI	2.991 (1.195-7.486)	0.019

OR, odds ratio; CI, confidence interval.

Adjust for: age, hypertension, diabetes mellitus, family history of cancer, and laterality of breast cancer.

## Discussion

Among breast cancer cases, LNM-positive patients are generally associated with a higher likelihood of recurrence and poorer survival outcomes (35). The identification of reliable indicators capable of effectively predicting LNM risk in breast cancer patients holds substantial clinical value for designing individualized therapeutic regimens and optimizing patient prognosis. This study focused on the association between the nutritional immune status of breast cancer patients and LNM. The results showed that a low PNI was significantly associated with LNM in breast cancer, while the HALP score had no statistical association with LNM in breast cancer. This finding not only provides new clinical evidence for potential influencing factors of LNM in breast cancer but also reveals the heterogeneity of different nutritional immune assessment indicators in the prognosis judgment of breast cancer.

The PNI, a classic composite indicator of nutritional and immune status, is calculated based on serum albumin (a marker of the body’s synthetic metabolic capacity) and peripheral blood lymphocyte count (reflecting cellular immune function). Its core value lies in integrating the assessment of the “nutritional reserve - immune defense” dual homeostasis. It reflects the synergistic effect of insufficient nutritional reserves and low immune function. During the progression of breast cancer, the rapid proliferation of tumors continuously depletes the body’s nutrients, and the lack of nutrition in turn inhibits the generation and activation of immune cells, forming a vicious cycle of nutritional depletion - immune suppression - tumor metastasis. Low PNI is precisely the external manifestation of this cycle. The results of this study are consistent with those of previous studies (36, 37). The HALP score is composed of hemoglobin (indicating oxygen supply and anemia status), albumin (a nutritional indicator), lymphocyte count (an immune indicator), and platelet count (a coagulation and inflammation indicator). It has been proven to be related to prognosis in some malignant tumors (29, 30, 38, 39). However, in this study, no association was found between it and LNM of breast cancer.

Albumin can inhibit tumor metastasis by regulating the tumor microenvironment (40, 41). When patients experience a decrease in albumin levels due to tumor consumption, insufficient intake, or liver dysfunction, on one hand, it will reduce the colloid osmotic pressure within the blood vessels, increasing the probability that tumor cells can penetrate the blood vessel wall and enter the lymphatic circulation (42). On the other hand, the low albumin state will reduce its binding with pro-inflammatory factors such as tumor necrosis factor-α (TNF-α) and interleukin-6 (IL-6), leading to an imbalance in the pro-inflammatory microenvironment (43). Pro-inflammatory factors can activate matrix metalloproteinases (MMPs) to degrade the extracellular matrix, creating conditions for tumor cells to break through the breast basement membrane and invade regional lymph nodes (44, 45). Hypoalbuminemia may create favorable conditions for LNM by impairing the body’s inhibitory effects on tumor cells and promoting tumor angiogenesis (46, 47).

Peripheral blood lymphocyte count directly reflects the cellular immune function. Among them, CD8+ cytotoxic T cells and natural killer (NK) cells can directly kill tumor cells by recognizing tumor antigens (48, 49), while helper T cells (CD4+) can regulate the immune response by secreting cytokines (50). When lymphocytes are reduced, the host’s immune surveillance against tumor cells is weakened. Tumor cells can evade immune recognition (by down-regulating MHC-I molecule expression) and induce immunosuppression (by recruiting regulatory T cells), proliferating in local tissues and migrating to lymph nodes (51, 52). Additionally, the low lymphocyte state will also reduce the secretion of anti-tumor cytokines such as interferon-γ (IFN-γ), and the insufficient expression of IFN-γ can inhibit tumor angiogenesis and lymphangiogenesis (53). Its deficiency will further promote the activation of the tumor lymphatic metastasis pathway. Notably, our study simultaneously evaluated the prognostic value of PNI and HALP, two complementary systemic inflammation−nutritional biomarkers. From a biological perspective, both indices reflect the complex interactions between systemic inflammation, immune suppression, malnutrition, and tumor progression, which collectively shape the tumor microenvironment and affect patient survival. Although these indices are constructed from overlapping components including albumin and lymphocytes, they reflect distinct dimensions of host physiological status. PNI primarily represents nutritional reserve and adaptive immune function, whereas HALP further incorporates hemoglobin and platelets, thereby comprehensively capturing systemic inflammation, anemia, malnutrition, and thrombotic tendency simultaneously.

The hemoglobin level mainly regulates metastasis by influencing the oxygen partial pressure in the tumor microenvironment (54). In some cancers, hypoxia in tumor tissues significantly induces the expression of hypoxia-inducible factor 1α (HIF-1α), thereby promoting angiogenesis and metastasis (55, 56). However, breast cancer has lower sensitivity to hypoxia, and its metastasis is more dependent on hormonal signals, and HER2 pathways, rather than simply the oxygen supply status (57). Additionally, the association between platelet count and tumor metastasis is mainly promoted by platelet-tumor cell aggregates for the survival of circulating tumor cells (58), but the expression level of platelet adhesion receptors (such as GPIIb/IIIa) on the surface of breast cancer cells is lower than that in gastric cancer, pancreatic cancer, etc., resulting in a weaker promoting effect of platelets on its metastasis (59). In addition, it may be partially attributed to the high biological heterogeneity of breast cancer and complex interactions between platelets and tumor biology (60). Different molecular subtypes of breast cancer each have their own unique tumor microenvironment, inflammatory state and metabolic characteristics (61). These differences may lead to inconsistent associations of hemoglobin, albumin, lymphocytes, and platelets with clinical outcomes across subtypes, thereby weakening the overall prognostic value of the combined HALP score. Future studies are warranted to perform stratified analyses according to molecular subtypes and explore whether the HALP score is related to the prognosis of breast cancer with different molecular subtypes.

The impact of the indicators in HALP on LNM in breast cancer is limited, ultimately leading to an overall lack of correlation in the HALP score. In breast cancer, the importance of different indicators varies. For instance, albumin and lymphocyte count have a much greater impact on breast cancer metastasis than hemoglobin and platelets. But in the HALP score, the latter two indicators are included and given equal weights, which may dilute the effect of the key indicators and prevent it from effectively capturing the association between nutritional immune status and LNM. In contrast, PNI focuses on the two indicators of albumin and lymphocytes, which are more crucial for breast cancer metastasis, and can more accurately reflect the risk. This also suggests that for different tumor types, the combination and weights of nutritional immune indicators need to be optimized rather than directly applying a universal scoring system.

Notably, PNI is closely associated not only with LNM but also with overall survival, prognosis, and adverse clinical outcomes in various malignant tumors (62). In the present study, subgroup analyses stratified by molecular subtypes were performed to verify the robustness of our primary findings. Consistent with the overall results, PNI was significantly associated with LNM in Luminal A, HER2+ and TNBC patients, and showed a borderline significant association in the Luminal B subtype, suggesting that PNI may serve as a stable and promising indicator for evaluating LNM risk across most molecular subtypes of breast cancer. In contrast, no significant correlation was observed between HALP and LNM in any of the four molecular subtypes, indicating that HALP might not be an effective marker for predicting LNM regardless of breast cancer molecular classification. These subgroup results further consolidate the reliability of our main conclusion that PNI is superior to HALP in identifying LNM in breast cancer.

This study clarifies the potential value of the PNI score in assessing the risk of LNM in breast cancer. Compared with other complex molecular biological indicators, the PNI confers advantages of convenient detection and low cost. Thus, it is expected to serve as a routine auxiliary indicator for screening metastasis risk in breast cancer patients. However, this study has certain limitations. First, as a retrospective study, it may be subject to selection bias, and the limited sample size may compromise the generalizability of the results. Second, the ROC analysis demonstrated that the AUC values of PNI and HALP were both approximately 0.61, suggesting relatively low discriminative ability for predicting the studied outcomes. PNI may serve as convenient, non-invasive, and cost-effective biomarkers for preliminary risk stratification, but they are not sufficient to be used as standalone diagnostic or high-precision predictive tools. Future studies with larger sample sizes, combined with other clinical, pathological, or molecular biomarkers, are warranted to improve the predictive accuracy and clinical utility of these scoring systems. Third, this study was conducted over a relatively long period from 2017 to 2024. During this time, surgical techniques, perioperative management protocols, and adjuvant therapies may have gradually improved. We did not perform specific statistical adjustments to account for these temporal changes and potential secular trends, which may introduce minor confounding effects.

The findings of this study highlight the potential clinical utility of the PNI as a simple and convenient preoperative biomarker for predicting LNM in breast cancer. From a clinical perspective, preoperative PNI assessment may provide valuable information for surgical decision−making and perioperative management. Patients with low PNI, representing compromised nutritional and immune status, may require more intensive perioperative nutritional support and immunomodulatory interventions to reduce surgical risk and improve postoperative recovery. Furthermore, identification of patients at high risk of LNM based on PNI may help guide individualized surgical planning and adjuvant treatment strategies. Future studies are warranted to validate whether routine preoperative evaluation of PNI can optimize clinical decision−making, refine perioperative care, and ultimately improve long−term oncologic outcomes in patients with breast cancer.

## Conclusions

This study has confirmed that a low PNI is a potential risk factor for LNM in breast cancer. Clinically, PNI can be used as a convenient warning tool for LNM in breast cancer, and attention should be paid to nutritional and immune intervention before and after surgery. The mechanism may be closely related to the weakened metabolic barrier caused by the decrease in albumin levels and the disintegration of the immune defense line triggered by the reduction in lymphocyte count. However, the HALP score is not associated with LNM in breast cancer, which may be due to the mismatch between the biological characteristics of breast cancer and the HALP indicators, the uniformity of the indicator distribution in the study population, and the defect in the score weight. In the future, multi-center prospective studies and mechanism experiments are needed to further verify the clinical value of PNI and provide new strategies for the precise prevention and treatment of breast cancer.

## Data Availability

The original contributions presented in the study are included in the article/supplementary material. Further inquiries can be directed to the corresponding author.
